# Brief Report: Preliminary Evidence of the N170 as a Biomarker of Response to Treatment in Autism Spectrum Disorder

**DOI:** 10.3389/fpsyt.2021.709382

**Published:** 2021-06-29

**Authors:** Shashwat Kala, Max J. Rolison, Dominic A. Trevisan, Adam J. Naples, Kevin Pelphrey, Pamela Ventola, James C. McPartland

**Affiliations:** ^1^Child Study Center, Yale School of Medicine, New Haven, CT, United States; ^2^Department of Neurology, University of Virginia, Charlottesville, VA, United States

**Keywords:** autism spectrum disoder, electroencephalography, N170, biomarker, pivotal response treatment

## Abstract

**Background:** Autism Spectrum Disorder (ASD) is a neurodevelopmental disorder characterized by primary difficulties in social function. Individuals with ASD display slowed neural processing of faces, as indexed by the latency of the N170, a face-sensitive event-related potential. Currently, there are no objective biomarkers of ASD useful in clinical care or research. Efficacy of behavioral treatment is currently evaluated through subjective clinical impressions. To explore whether the N170 might have utility as an objective index of treatment response, we examined N170 before and after receipt of an empirically validated behavioral treatment in children with ASD.

**Method:** Electroencephalography (EEG) data were obtained on a preliminary cohort of preschool-aged children with ASD before and after a 16-week course of PRT and in a subset of participants in waitlist control (16-weeks before the start of PRT) and follow-up (16-weeks after the end of PRT). EEG was recorded while participants viewed computer-generated faces with neutral and fearful affect.

**Results:** Significant reductions in N170 latency to faces were observed following 16 weeks of PRT intervention. Change in N170 latency was not observed in the waitlist-control condition.

**Conclusions:** This exploratory study offers suggestive evidence that N170 latency may index response to behavioral treatment. Future, more rigorous, studies in larger samples are indicated to evaluate whether the N170 may be useful as a biomarker of treatment response.

## Introduction

Autism spectrum disorder (ASD) is a heterogeneous neurodevelopmental disorder hallmarked by difficulties with social communication, along with restricted and repetitive behaviors and atypical response to sensory information (1). Without objective biomarkers for ASD, clinical practice and research are reliant on subjective clinician judgments. There is a critical need to identify objective biomarkers for ASD to enhance clinical research by providing quantifiable indices of functional processes relevant to ASD, such as face perception.

The N170 is a well-studied neural marker of face perception. This face-sensitive event-related potential (ERP) is evident as a negative deflection over occipitotemporal scalp ~170 milliseconds (ms) after viewing a face and indexes structural encoding, an early stage of face processing (2). The latency of the N170 reflects temporal processing of faces, such that longer latencies reflect slower, less efficient face processing or incomplete developmental maturation. Delayed N170 latency is observed in individuals with ASD relative to age- and IQ-matched typically developing (TD) children (3), a neuroscientific finding that is reproducible across heterogeneous ASD samples (4). Right hemisphere N170 latency to upright faces was recently accepted into the FDA Center for Drug Evaluation and Research (CDER) Biomarker Qualification Program, making it the first biomarker for a psychiatric disease to receive this designation (5).

Most N170 studies to date have focused on establishing group mean differences and relationships with symptomatology. These features are germane to multiple biomarker contexts of use, such as stratification into treatment-relevant subgroups; however, they provide limited information regarding its potential utility in other desired contexts of use, such as quantifying change in neural systems in response to treatment. Determination of viability in this context of use requires appropriately designed studies that measure N170 latency in children with ASD in the context of intervention and associated change in clinical status.

Very few studies have examined N170 latency as a potential index of treatment response. Dawson et al. (6) examined neural correlates of face perception subsequent to early behavioral intervention in 48- to 77-month-old children. Though differences in the Nc, an attention-related ERP arising from the prefrontal and anterior cingulate cortices (7), in response to faces was observed between intensive intervention and community treatment groups, significant differences were not observed at the N170. This study indicates the appropriateness of face processing circuitry for quantifying response to treatment. It is, however, difficult to draw conclusions regarding the appropriateness of the N170 as a biomarker of treatment response from these data given the absence of pre-intervention EEG recordings and the consequent inability to conduct within-participant comparisons of N170 change in response to treatment. A second study examined changes in N170 in response to a drama-based social skills group intervention and failed to detect changes associated with treatment relative to a waitlist control (8). Given evidence that social skills groups, administered at a lower intensity level than individualized behavioral treatments, do not consistently improve face perception [(9); but see (10)], it is possible that the systems indexed by the N170 were not affected by this treatment. Conclusions regarding the potential utility of the N170 in this study are also complicated by the observed improvements on measures of face perception and shorter N170 latencies at posttest relative to baseline, suggesting placebo effects could have obscured associations with change in N170.

The current study sought to explore the potential utility of N170 latency as an index of treatment response in a preliminary study designed to address several of the limitations of prior research. Like previous studies, we examined children over the course of receipt of an empirically validated intensive and individualized intervention, in this case, pivotal response treatment [PRT; (11)]. To build on prior research and potentially improve sensitivity to evaluate change in a neural biomarker, we: (1) collected pre- and post-test data to permit intra-individual comparisons; (2) administered treatment individually rather than in a group setting and focused specifically on social-communication; (3) administered treatment over an extended period of time (16 weeks) and with a high level of intensity to increase the likelihood of changing neural systems; (4) utilized a treatment already demonstrated to enact change in social perceptual brain systems (12).

We hypothesized that children would exhibit behavioral improvement in response to PRT and that right hemisphere N170 latency, commonly increased in ASD relative to TD children, would decrease in response to treatment. In contrast, we predicted that the P100, a positive-going component arising from the parieto-occipital region ~100 ms after stimulus presentation and reflecting low-level visual processing (13, 14), would not be affected by social-communicative treatment.

## Methods

### Participants

Seven 4- to 7-year-old children with ASD received a 16-week course of PRT as part of an ongoing research study at the Yale School of Medicine (Table 1). Of these seven participants, three served in a waitlist control condition prior to enrolling in treatment, and five served in a follow-up condition conducted 16 weeks after the end of PRT. All study participants met gold-standard diagnostic criteria for ASD according to the Autism Diagnostic Observation Schedule [ADOS; (15)] and the Autism Diagnostic Interview-Revised [ADI-R; (16)] and had IQs > 70. PRT targeted social communication skills and play for 8 hours per week, which involved direct work with the child and parent in clinic and at home.

**Table 1:** Participant information at the pre-PRT timepoint.

**Participant**	**Age (years)**	**DAS**	**ADOS total**	**SRS T-score**	**VABS socialization domain standard score**
1	5.78	127	12	68	95
2	7.01	95	26	70	80
3	5.16	121	24	68	88
4	4.51	122	11	61	88
5	6.35	106	19	61	97
6	5.48	110	11	78	81
7	4.59	105	22	72	85
Mean	5.55	112.3	17.9	68.3	87.7

### EEG Recording Procedure

EEG data collection was attempted at four time points: 16 weeks prior to the start of treatment (for the waitlist control group only), pre-treatment, post-treatment, and 16 weeks after the conclusion of treatment (follow-up). Participants were included in analysis if they contributed good quality data for both pre- and post-treatment visits. Thus, fewer participants have data for the waitlist control and follow-up EEG sessions.

The EEG paradigm consisted of 70 computer-generated, grayscale faces (35 male and 35 female) displaying neutral and fearful affect. Participants viewed 146 dynamic trials in random sequence, lasting a total of 15 minutes. There were 70 neutral to fearful faces, 70 fearful to neutral faces, and a total of 6 targets to maintain attention (17).

**Figure 1:**
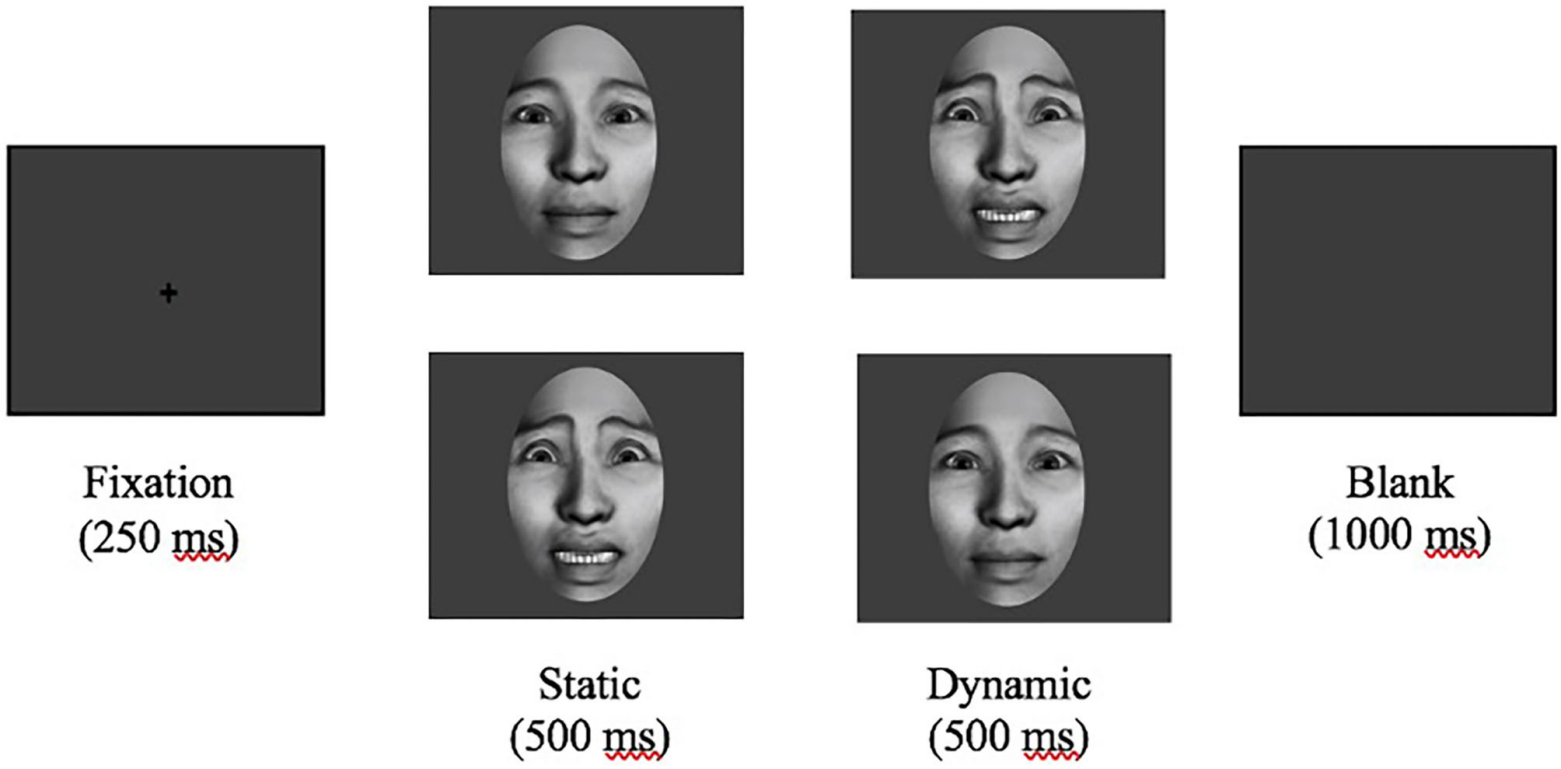
Experimental paradigm. After a 200–300 ms fixation crosshair, a static neutral or fearful face was presented for 500 ms before dynamically changing expression. ERP data was segmented to an epoch 100 ms before to 500 ms after presentation of the initial static face.

Each trial consisted of a central fixation crosshair presented for 200–300 ms followed by a static face with either a neutral or fearful expression appearing on the center of the screen for 500 ms. Afterward, the face changed from either neutral to fearful or fearful to neutral expression in an animated, realistic movement. This second face was also presented on the center of the screen for 500 ms (Figure 1, Supplementary Material 1). In total, faces were presented for 1,000 ms for each trial. During the paradigm, participants were instructed to press a button in response to a target stimulus, white balls, interspersed throughout the paradigm to maintain attention. A behavioral assistant was seated with all participants throughout EEG recording to monitor attention and limit participant movement.

### EEG Analysis

**Figure 2:**
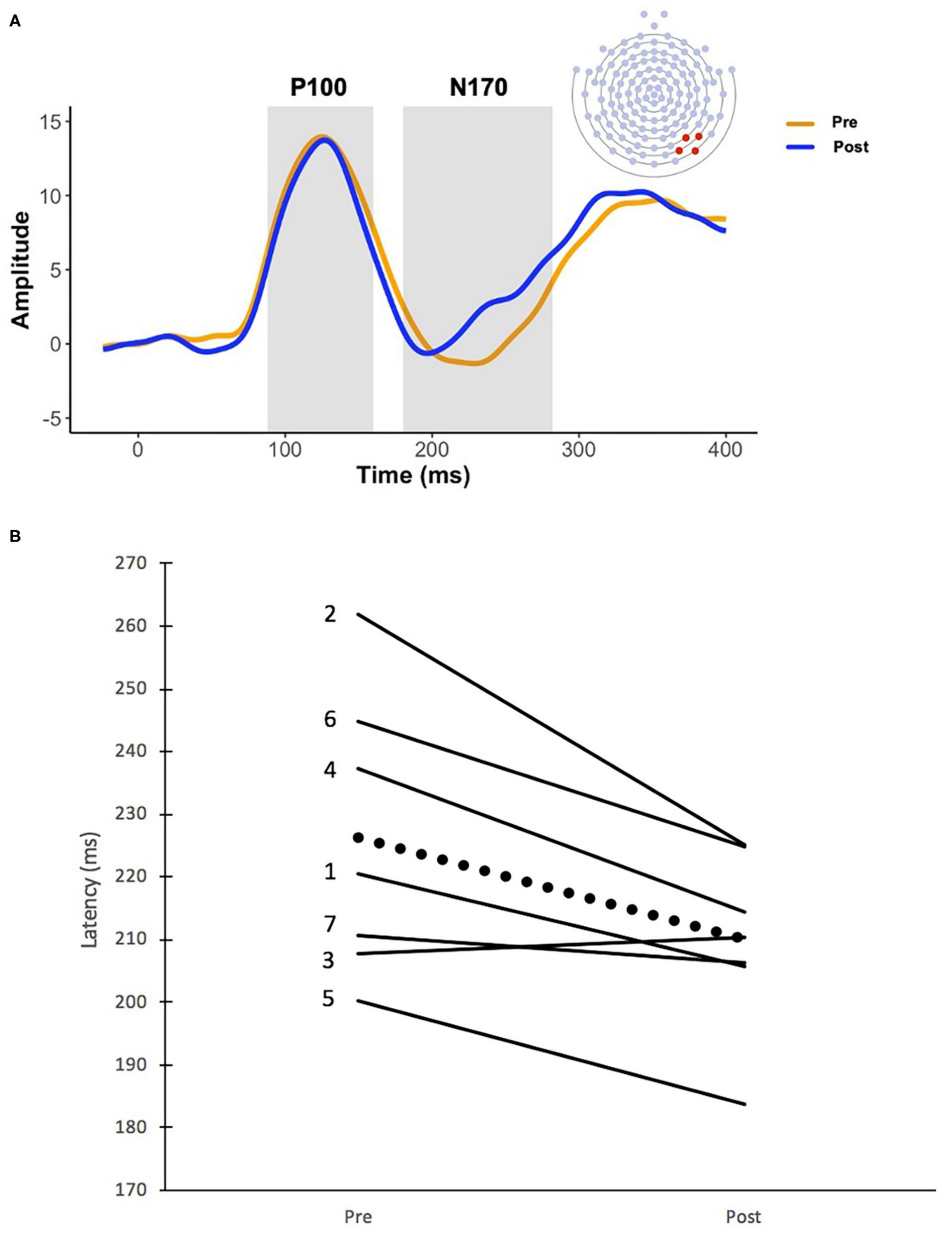
**(A)** Grand averaged waveforms before and after 16 weeks of PRT. The orange line represents the recording prior to PRT and the blue line represents the recording after PRT. Temporal windows for the P100 (88–160 ms) and the N170 (180–282 ms) are represented by the gray boxes, and the P100 and N170 components are labeled accordingly. N170 electrode recording sites are also depicted. ERP data were averaged over the right occipitotemporal region (electrodes 89, 90, 94, 95). **(B)** Changes in N170 latency before and after 16 weeks of PRT. Solid lines indicate individual change in N170 latency for each of the seven subjects and the dotted line represents average group change. Numbers to the left of each line correspond with participant numbers listed in Table 1.

EEG was recorded with a 128-channel Geodesic sensor net. Data was analyzed offline using NetStation 4.5.4. Data was filtered at 0.1–30 Hz and then segmented to an epoch 100 ms before to 500 ms after presentation of the initial static face. Data was baseline corrected to the 100 ms preceding stimulus presentation and re-referenced to an average. Trials with eye movements and blinks were detected and excluded using NetStation's eyeblink and eye-movement algorithms (±100 μV threshold for eye movements and ±140 μV for eye blinks). Channels were marked bad in each trial if they exceeded 200 μV for the entire trial and based on visual inspection. If channels were marked bad in more than 40% of trials, the channel was marked bad for all remaining trials. If a trial contained more than 10 bad channels (>15%), eye blinks, or other eye movements, it was excluded from further analysis. If a trial contained fewer than 10 bad channels, the bad channels were replaced using spherical spline interpolation (18). Trial by trial data were averaged at each electrode for the fear and neutral conditions for each participant. Participants were required to have 15 good trials per condition to be included for analyses, so all included participants had at least 30 adequate trials. ERP data were averaged over the right occipitotemporal region [(19); electrodes 89, 90, 94, 95; Figure 2A], consistent with previous research showing that neural regions specialized for face perception, namely the fusiform face area and superior temporal sulcus, are lateralized to the right hemisphere (20). Temporal windows for the P100 and N170 were 88–160 ms and 180–282 ms, respectively, based on maximal amplitude in grand averages and confirmed in individual averages. Latency and amplitude of the maximal peak within the 88–160 ms window were extracted for the P100. Latency and amplitude of the minimal peak within the 180–282 ms window were extracted for the N170.

### Statistical Analysis

P100 amplitude, P100 latency, N170 amplitude, and N170 latency were analyzed using four separate repeated measures analysis of variance (ANOVA) with pre-treatment and post-treatment ERP components as within-subject factors. N170 latencies to neutral and fearful faces were comparable pre- and post-PRT (*p* = 0.97, *p* = 0.35), as were N170 amplitudes (*p* = 0.70, *p* = 0.58). Similarly, P100 latencies to neutral and fearful faces were comparable pre- and post-PRT (*p* = 0.58, *p* = 0.21), as were P100 amplitudes (*p* = 0.59, *p* = 0.29). As a result, fearful and neutral conditions were collapsed at each time point for each ERP component. When significant, follow-up repeated measures ANOVAs were used to compare pre-ERP data to the waitlist control subset and post-ERP data to the follow-up condition subset. Additionally, changes in clinical symptomatology reflected in total ADOS scores (15), overall Social Responsiveness Scale (SRS) T-scores (21), and the socialization domain of the Vineland Adaptive Behavior Scales [VABS; (22)] were analyzed using repeated measures ANOVAs. When applicable, the relationships between changes in ERP data and changes in clinical symptomatology were analyzed using Pearson's correlations.

## Results

### Behavioral Measures

Children participating in treatment displayed significant reductions in total ADOS scores, indicating an improvement in clinical symptomatology [*F*_(1, 6)_ = 12.67, *p* = 0.012, $\eta_{\text{p}}^{2}$ = 0.679]. Significant changes in SRS T-scores [*F*_(1, 6)_ = 1.20, *p* = 0.315, $\eta_{\text{p}}^{2}$ = 0.167], the VABS socialization domain [*F*_(1, s6)_ = 1.08, *p* = 0.340, $\eta_{\text{p}}^{2}$ = 0.152], and socialization subdomains (*p*s > 0.05) were not observed.

### ERP Results

#### P100

No significant change was observed in either P100 latency [*F*_(1, 6)_ = 0.88, *p* = 0.384, $\eta_{\text{p}}^{2}$ = 0.128] or P100 amplitude [*F*_(1, 6)_ = 0.037, *p* = 0.854, $\eta_{\text{p}}^{2}$ = 0.006] between pre- and post- time points.

#### N170

A main effect of time point indicated a significant reduction in N170 latency after PRT [*F*_(1, 6)_ = 11.18, *p* = 0.016, $\eta_{\text{p}}^{2}$ = 0.651], with average N170 latency decreasing from 226 ms (SD = 22.48 ms) to 210 ms (SD = 14.05 ms) (Figures 2A,B). There was no significant change in N170 amplitude before and after treatment [*F*_(1, 6)_ = 2.70, *p* = 0.152, $\eta_{\text{p}}^{2}$ = 0.310].

### Waitlist and Follow-Up

Given statistically significant decreases in N170 latency between pre- and post-treatment, exploratory analyses compared differences with the waitlist (*n* = 3) and follow-up (*n* = 5) subgroups. There was no significant change in N170 latency in the 16-week period from the waitlist condition to the start of PRT [*F*_(1, 2)_ = 2.50, *p* = 0.255, $\eta_{\text{p}}^{2}$ = 0.556] and also no change in N170 latency in the 16-week period from the end of PRT to the follow-up condition [*F*_(1, 4)_ = 5.48, *p* = 0.079, $\eta_{\text{p}}^{2}$ = 0.578].

### Relationship Between N170 and Behavioral Measures

No significant correlations between electrophysiological changes in N170 latency and changes in total ADOS scores (*r* = −0.275, *p* = 0.551), SRS scores (*r* = −0.314, *p* = 0.493), Vineland socialization domain scores (*r* = −0.165, *p* = 0.723), or socialization subdomain scores (*p*s > 0.05) were found.

## Discussion

Consistent with our hypotheses, significant reductions in N170 latency were observed in 4- to 7-year-old children with ASD receiving a 16-week course of PRT. Neural changes were specific to N170 latency and were not observed in N170 amplitude or P100 latency and amplitude. This pattern of results suggests that face processing efficiency, rather than basic visual processing of low-level features of visual stimuli, was selectively impacted by PRT. As predicted based on extensive prior evidence, PRT treatment was associated with reductions in autism symptomatology, paralleling changes observed in N170 latency. In our small sample, correlations between magnitude of neural and behavioral change were not observed. Exploratory analyses in subgroups suggest stability of these changes in N170 latency during a 16-week follow-up period after treatment. Similarly, the meaningfulness of changes observed during treatment is supported by stability in the 16 weeks preceding treatment. This pattern of results suggests repeated administration of the experimental assay alone does not lead to N170 change.

These findings offer suggestive evidence of the potential of the N170 as a biomarker sensitive to change in clinical status in the context of intervention. This is an important prospect in several regards. These findings align with prior results using fMRI (12); by extending these findings to EEG, we demonstrate the potential utility of a more economical, scalable, developmentally accessible, and tolerable technology (23) for quantifying neural change in response to treatment. The potential value of a direct measurement of central nervous system change in treatment is significant. All treatments, behavioral or pharmacological, necessarily exert their actions on the brain; objective quantifications of change at the neural level hold potentially greater sensitivity than subjective clinical measures of downstream behavior. In this way, biomarkers could indicate effectiveness in a shorter time scale or with greater sensitivity than the caregiver and clinician rating scales that represent the *status quo* (24).

### Limitations and Future Directions

This exploratory study has significant limitations, most notably its small size and cognitively-able sample. This participant profile limits the generalizability of our findings to the ASD community at large, but the detection of significant effects despite limited statistical power is salient and suggests the value of replication in larger, heterogeneous samples. Though we observed sensitivity to change that paralleled behavioral change, change in biomarker values did not correlate with clinical change. Such correlations would provide stronger evidence of convergent validity and should be re-examined in larger samples with a potentially greater range of change, more granular content in clinical measures, and inclusion of behavioral metrics of face perception. Additionally, this study did not include a non-face stimulus to establish the specifity of effects to social perception. Although this was purposeful to maximize tolerability of the paradigm for young children with ASD, future studies should evaluate the possibility that behavioral intervention improves non-social aspects of visual perception. We note that the absence of observed change at the P100 is supportive of our interpretation of the treatment effects being specifically relevant to social perception. Though we included an attention task and had a behavioral assistant monitor participant attention, rigor would be enhanced by future studies including eye tracking to monitor attention.

These preliminary findings suggest the value of continued investigation of the potential of the N170 as a biomarker in contexts of use related to quantification of treatment response.

## Data Availability Statement

The raw data supporting the conclusions of this article will be made available by the authors, without undue reservation.

## Ethics Statement

The studies involving human participants were reviewed and approved by the Human Investigation Committee at Yale School of Medicine consistent with the 1964 Declaration of Helsinki. Written informed consent to participate in this study was provided by the participants' legal guardian/next of kin.

## Author Contributions

JM, AN, KP, and PV contributed to study conception and design. EEG acquisition was performed by MR and other members of JM's research team. EEG analysis was completed by MR. MR, DT, AN, and SK performed statistical analyses. PV and her team administered intervention. The manuscript was written by SK and MR. All authors contributed to and approved the final manuscript.

## Conflict of Interest

JM consults with Customer Value Partners, Bridgebio, Determined Health, and BlackThorn Therapeutics, has received research funding from Janssen Research and Development, serves on the Scientific Advisory Boards of Pastorus and Modern Clinics, and receives royalties from Guilford Press, Lambert, and Springer. The remaining authors declare that the research was conducted in the absence of any commercial or financial relationships that could be construed as a potential conflict of interest.
