# Supplementary material for: Brief Report: Preliminary Evidence of the N170 as a Biomarker of Response to Treatment in Autism Spectrum Disorder
Source: Front Psychiatry. 2021 Jun 29;12:709382. doi: 10.3389/fpsyt.2021.709382 (PMC8275957; doi:10.3389/fpsyt.2021.709382)
Supplement: Supplementary file 1 [file Table_1.DOCX]

Supplement 1:

Sample stimuli are available to view at <https://medicine.yale.edu/lab/mcpartland/publications/>
